# Economic evaluation of a behavioral intervention versus brief advice for substance use treatment in pregnant women: results from a randomized controlled trial

**DOI:** 10.1186/s12884-017-1260-5

**Published:** 2017-03-07

**Authors:** Xiao Xu, Kimberly A. Yonkers, Jennifer Prah Ruger

**Affiliations:** 1 Department of Obstetrics, Gynecology and Reproductive Sciences, Yale School of Medicine, 310 Cedar Street, LSOG 205B, New Haven, CT 06520 USA; 2 Department of Psychiatry, Yale School of Medicine, New Haven, CT USA; 3 Department of Medical Ethics and Health Policy, Perelman School of Medicine, The Leonard Davis Institute of Health Economics, University of Pennsylvania, Philadelphia, PA USA

**Keywords:** Cost minimization analysis, Economic evaluation, Substance use, Pregnancy, Randomized controlled trial

## Abstract

**Background:**

Substance use in pregnancy is associated with severe maternal and fetal morbidities and substantial economic costs. However, few studies have evaluated the cost-effectiveness of substance use treatment programs in pregnant women. The purpose of this study was to evaluate the economic impact of a behavioral intervention that integrated motivational enhancement therapy with cognitive behavioral therapy (MET-CBT) for treatment of substance use in pregnancy, in comparison with brief advice.

**Methods:**

We conducted an economic evaluation alongside a clinical trial by collecting data on resource utilization and performing a cost minimization analysis as MET-CBT and brief advice had similar effects on clinical outcomes (e.g., alcohol and drug use and birth outcomes). Costs were estimated from the health care system’s perspective and included intervention costs, hospital facility costs, physician fees, and costs of psychotropic medications from the date of intake assessment until 3-month postpartum. We compared effects of MET-CBT on costs with those of brief advice using Wilcoxon rank sum tests.

**Results:**

Although the integrated MET-CBT therapy had higher intervention cost than brief advice (median = $1297/participant versus $303/participant, *p* < 0.01), costs of care during the prenatal period, delivery, and postpartum period, as well as for psychotropic medications, were comparable between the two groups (all *p* values ≥ 0.55). There was no statistically significant difference in overall cost of care (median total cost = $26,993/participant for MET-CBT versus $27,831/participant for brief advice, *p* = 0.90).

**Conclusions:**

The MET-CBT therapy and brief advice resulted in similar clinical outcomes and overall medical costs. Further research incorporating non-medical costs, targeting women with more severe substance use disorders, and evaluating the impact of MET-CBT on participants’ quality of life will provide additional insights.

**Trial registration:**

ClinicalTrials.gov NCT00227903. Registered 27 September 2005.

## Background

Use of illicit drugs, alcohol and tobacco during pregnancy is associated with severe maternal and fetal morbidities, such as placental abruption, birth defects, preterm birth, drug withdrawal syndromes and longer-term developmental problems [1–4]. Illicit drug use by pregnant women also increases their risk for human immunodeficiency virus (HIV) infection and subsequent risk for vertical transmission to infants [1, 5].

The rate of substance use in pregnancy is high and it is associated with substantial economic costs. National survey data showed that in the U.S., 5.9, 8.5 and 15.9% of pregnant women 15–44 years of age reported current use of illicit drugs, alcohol and cigarettes, respectively, in 2011–2012 [6]. Treatment for opioids-exposed newborns cost $70.6-$112.6 million in the U.S. in 2009 [7], and the average lifetime cost for a newborn with fetal alcohol syndrome was estimated to be $2.0 million in 2002 [8].

Although pregnant substance users have unique medical, psychological and social care needs, few studies have evaluated substance use treatment programs tailored to pregnant women and the limited number of studies available have targeted particular substances, such as nicotine or alcohol alone [9, 10], rather than women who use a variety of substances which is common in pregnant substance users [11]. Moreover, literature on the economic impact of these interventions is sparse [12], despite growing concerns about health care spending. Their clinical effectiveness needs to be carefully weighed against cost consequences in order to deliver high-value care.

In this study, we conducted an economic evaluation of an innovative intervention integrating motivational enhancement therapy with cognitive behavioral therapy (MET-CBT), in comparison with brief advice, designed for reducing substance use and HIV risk behaviors in pregnant women who use an array of substances. The findings will inform future allocation of resources for addressing substance use in pregnancy.

## Methods

### Intervention and participants

This study was an economic evaluation alongside a randomized controlled trial (RCT), i.e., Therapeutic Substance Abuse Treatment in Pregnancy-1 (PRIDE-P) trial. Details about the design, intervention, methods, and main clinical outcomes of the PRIDE-P trial are reported elsewhere [13]. Briefly, an MET-CBT intervention was designed to reduce substance use in pregnancy, decrease risky sexual behaviors, and improve birth outcomes. Pregnant women who used substances (alcohol, marijuana, cocaine, or stimulants but not solo use of opiates or nicotine) during the 28 days prior to screening or scored at least a “3” on the modified TWEAK (Tolerance, Worried, Eye-openers, Amnesia, K[C] Cut Down) screening test were eligible if they were 16 years of age or older and less than 28 weeks of gestation at the time of screening [13–15]. Patients whose primary substance was opioid or nicotine were excluded because methadone maintenance programs are the established treatment for patients with opioid use disorders and the study sites already had standard nicotine treatment protocols at the time of this study. Participants were randomly assigned to receive either the MET-CBT intervention or brief advice.

Women assigned to the MET-CBT group received one-on-one therapy from a trained nurse therapist at prenatal visits with optional booster visits after delivery (average number of sessions completed = 5.2). Each session lasted for about 30 min. Topics covered included: motivational enhancement, functional analysis (non-drug activities and triggers/patterns), safe sexual behavior, communication skills, relapse prevention, and problem-solving skills.

For participants assigned to receive brief advice, their routine obstetric providers provided brief guidance about the risks of substance use, importance of abstinence, and benefit of seeking drug and alcohol treatment outside of the prenatal setting (average number of sessions = 7.2). This discussion occurred within the routine prenatal/postpartum visits and each lasted for about 1 min.

A total of 168 study participants were recruited from two hospital-based reproductive health clinics between June 2006 and July 2010 [13]. Only one study site was able to provide complete cost data; hence, our analysis focused on the subset of participants who received prenatal care and delivered at this site in order to perform a comprehensive economic evaluation. This resulted in an analytical sample size of 112 participants, of which 60 were randomized to MET-CBT and 52 received brief advice.

### Clinical effectiveness

Results on clinical effectiveness of the integrated MET-CBT therapy, relative to brief advice, in the PRIDE-P trial are detailed elsewhere [13]. Briefly, participants were assessed at baseline, delivery, and 3-month postpartum. The primary outcome measure for clinical effectiveness was the percentage of days that a participant used alcohol or any drug during the prior 28 days [13, 16]. Secondary outcomes included whether the participant was abstinent from substances (alcohol and drugs) according to self-report, urine toxicology test, or combined self-report and urine toxicology test, respectively; whether she was abstinent from alcohol according to breathalyzer tests; the Addiction Severity Index (ASI) [17] composite scores for alcohol and drug use; participants’ sexual behaviors; and birth outcomes. Because there were no statistically significant treatment effects on primary or secondary clinical outcomes [13], the economic evaluation constituted a cost minimization analysis wherein, given similar effectiveness, we assessed which intervention was associated with lower costs.

### Cost

Economic evaluation was conducted from the health care system’s perspective. Measurement of costs included intervention costs, hospital facility costs, physician fees, and costs of psychotropic medications. Timeframe of the cost analysis began from the date of intake assessment until 3-month postpartum. Because this timeframe was less than 1 year, no discounting was needed. Intervention costs were determined using a micro-costing technique taking into consideration the quantity and unit price of all inputs used for the design and implementation of the MET-CBT intervention and brief advice [18]. Research-induced costs during the intervention (e.g., staff time spent on data entry) were excluded. Data on hospital facility costs were obtained from the institutional accounting database and therefore reflected actual medical resources consumed by participants and their newborns at each encounter (rather than charges or reimbursements). Physician costs were estimated based on actual payments received using data from a billing database for professional fees at the study site. Collectively, these hospital facility costs and physician costs encompassed all medical care services received by participants and their newborns at the study site from intake assessment until 3-month postpartum, including pregnancy-related as well as other medical services. To help elucidate the economic impact of the study interventions by phase of care, we categorized costs according to the time period when they occurred (i.e., prenatal period, labor and delivery, and postpartum period) and stratified by care for the mother versus the newborn. In addition, we reviewed medical records and obtained information on name, dose, frequency and duration of psychotropic medications used by participants during the study period. Average wholesale prices from Red Book Drug Topics [19] were used to estimate the cost of medications. Costs occurring in different years were inflation adjusted to 2012 U.S. dollars using the medical care component of consumer price index [20].

### Statistical analysis

We used non-parametric Wilcoxon rank sum test for all comparisons of costs between MET-CBT and brief advice due to skewed distribution of cost data. Four participants had otherwise complete data but lacked information on physician cost for their labor and delivery hospitalization. For them, we applied a diagnosis-related group (DRG)-specific, average physician cost to hospital facility cost ratio (whenever feasible) or intervention group-specific average physician cost for labor and delivery based on the rest of the sample. Participants with other types of missing data (e.g., lacked data on physician costs entirely) were excluded from the primary analysis. In all statistical tests, *p* values less than 0.05 were considered statistically significant.

In a sensitivity analysis, we included participants with missing costs by imputing their values using sample average costs from their corresponding intervention group (MET-CBT versus brief advice), setting (physician cost, hospital facility cost, versus medication cost), and time period (prenatal, labor and delivery, versus postpartum) or based on DRG-specific, average physician cost to hospital facility cost ratio (when feasible). For missing data on costs of psychotropic medications, we imputed the value using intervention group-specific average medication cost. In addition, three participants had twin pregnancies. As these pregnancies were substantially more expensive which might unduly influence comparison of costs due to the relatively small sample size, we conducted another sensitivity analysis focusing on participants with singleton births only.

## Results

### Participant characteristics

Participants’ mean age was 25.1 years (standard deviation = 6.1) (Table 1). Over half (53.2%) were black, and 25.9% were Hispanic. Thirty-five percent had less than high school education, and most participants (94.6%) had Medicaid coverage. Marijuana was the most frequently reported primary substance, followed by alcohol and cocaine. Over half of the participants (51.8%) reported ever regularly using more than one substance, and 32.1% of the participants were determined as alcohol/drug abuse or dependent according to the Mini-International Neuropsychiatric Interview (MINI) [21]. Average gestation age at the time of enrollment was 20.1 weeks.

**Table 1:** Patient characteristics at baseline

Characteristics	Overall (*n* = 112)	Brief Advice (n = 60)	MET-CBT (*n* = 52)
Age (in years)	25.1 ± 6.1	25.4 ± 5.8	24.8 ± 6.4
Race			
White	36 (32.4%)	19 (32.2%)	17 (32.7%)
Black	59 (53.2%)	31 (52.5%)	28 (53.8%)
Other	16 (14.4%)	9 (15.3%)	7 (13.5%)
Hispanic ethnicity			
Yes	29 (25.9%)	17 (28.3%)	12 (23.1%)
No	83 (74.1%)	43 (71.7%)	40 (76.9%)
Education (in years)			
< 12	39 (35.1%)	20 (33.3%)	19 (37.3%)
12	42 (37.8%)	20 (33.3%)	22 (43.1%)
13–15	25 (22.5%)	16 (26.7%)	9 (17.7%)
≥ 16	5 (4.5%)	4 (6.7%)	1 (2.0%)
Type of insurance/payer			
Medicaid	106 (94.6%)	54 (90.0%)	52 (100.0%)
Other	6 (5.4%)	6 (10.0%)	0 (0.0%)
Parity			
0	42 (37.5%)	22 (36.7%)	20 (38.5%)
1	34 (30.4%)	21 (35.0%)	13 (25.0%)
≥ 2	36 (32.1%)	17 (28.3%)	19 (36.5%)
Gestation age at time of enrollment, weeks	20.1 ± 7.2	20.3 ± 7.3	20.0 ± 7.2
Primary drug			
Alcohol	36 (32.1%)	18 (30.0%)	18 (34.6%)
Cocaine	23 (20.5%)	12 (20.0%)	11 (21.2%)
Marijuana	44 (39.3%)	24 (40.0%)	20 (38.5%)
Other	9 (8.0%)	6 (10.0%)	3 (5.8%)
Ever regularly used more than one substance			
Yes	57 (51.8%)	33 (56.9%)	24 (46.2%)
No	53 (48.2%)	25 (43.1%)	28 (53.8%)
MINI drug/alcohol abuse/dependence			
Yes	36 (32.1%)	23 (38.3%)	13 (25.0%)
No	76 (67.9%)	37 (61.7%)	39 (75.0%)

*MET-CBT* motivational enhancement therapy with cognitive behavioral therapy, *MINI* Mini-International Neuropsychiatric Interview. Statistics were reported as n (%) or mean ± standard deviation. Percentages may not add up to 100% due to rounding

### Costs

Intervention cost was significantly higher for MET-CBT than for brief advice (median = $1297/participant versus $303/participant, *p* < 0.01) because of its higher set-up costs and longer session duration (Table 2) [18]. This difference, however, was overshadowed by the substantial medical costs that occurred during the period of pregnancy, delivery, and postpartum. The median per participant cost during prenatal, delivery and postpartum period was $6339, $15,493, and $1272, respectively, for participants receiving MET-CBT, compared with $6591, $15,175, and $1254, respectively, for participants receiving brief advice (all *p* values ≥ 0.55). Costs of psychotropic medications were also similar between the two groups (median = $124 versus $136, *p* = 0.70). Overall, there was no statistically significant difference in total cost of care for participants between the two groups (median = $26,993/participant in MET-CBT versus $27,831/participant in brief advice, *p* = 0.90).

**Table 2:** Comparison of per participant costs between brief advice and integrated motivational enhancement and cognitive behavioral therapy

Cost Category	Brief Advice (*n* = 58)		MET-CBT (*n* = 48)		*P* value
	Median	(Interquartile range)	Median	(Interquartile range)	
Intervention	$303	($264-$329)	$1297	($1178-$1491)	<0.01
Prenatal period	$6591	($4819-$9909)	$6339	($3436-$9977)	0.55
Labor and delivery					
Mother	$9399	($7664-$11,745)	$9516	($7101-$12,113)	0.91
Baby	$3611	($2410-$18,816)	$3708	($2219-$24,704)	0.88
Mother + Baby	$15,175	($11,194-$32,926)	$15,493	($10,352-$33,507)	0.94
3-month postpartum period					
Mother	$385	($225-$763)	$657	($258-$1196)	0.12
Baby	$757	($235-$1278)	$820	($394-$1275)	0.63
Mother + Baby	$1254	($775-$2434)	$1272	($818-$2782)	0.65
Medication	$136	($0-$194)	$124	($0-$187)	0.70
Total	$27,831	($19,694-$48,273)	$26,993	($21,240-$45,289)	0.90

six participants were excluded from primary analysis due to missing cost data. *MET-CBT* motivational enhancement therapy with cognitive behavioral therapy

**Fig. 1:**
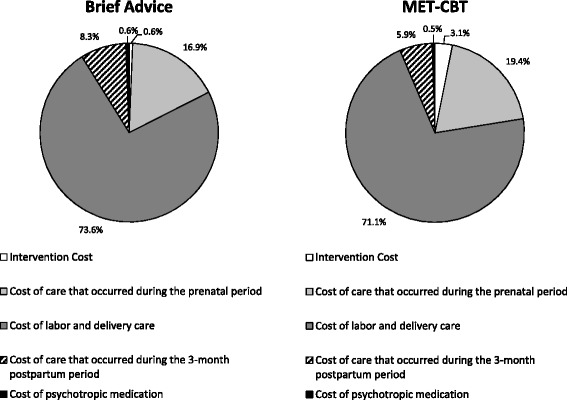
Distribution of cost categories. MET-CBT = motivational enhancement therapy with cognitive behavioral therapy. Percentages may not add up to 100% due to rounding

Distributions of costs were right skewed as a small number of participants incurred high costs. For example, 20 mothers (17.9%) stayed in hospital for longer than 4 days; and of the 114 newborns with known length of stay (including 3 pairs of twins), 24 (21.1%) were admitted for more than 10 days. As a result, mean overall cost was much higher than the median (mean = $43,294/participant and $47,693/participant for MET-CBT and brief advice, respectively). Maternal and newborn hospital stay for delivery accounted for the largest share of the overall cost (71.1% for the MET-CBT group and 73.6% for the brief advice group), followed by care received during the prenatal period (19.4 and 16.9% for MET-CBT and brief advice, respectively) (Fig. 1).

Similar results were found in sensitivity analyses when only singleton births were included and when participants with missing data were included with imputed values. Median per participant cost was $26,817 for MET-CBT versus $27,537 for brief advice (*p* = 0.82) and $26,314 for MET-CBT versus $27,537 for brief advice (*p* = 0.97), respectively (Table 3).

**Table 3:** Sensitivity analyses of per participant costs between brief advice and integrated motivational enhancement and cognitive behavioral therapy

	Brief Advice		MET-CBT		*P* value
Analysis	Median	(Interquartile Range)	Median	(Interquartile Range)	
Base case analysis	$27,831	($19,694-$48,273)	$26,993	($21,240-$45,289)	0.90
Limiting to singleton births	$27,537	($19,354-$45,131)	$26,817	($21,070-$44,172)	0.82
Including participants with missing data (via imputation)	$27,537	($18,891-$47,519)	$26,314	($20,538-$43,914)	0.97

*MET-CBT* motivational enhancement therapy with cognitive behavioral therapy

## Discussion

Drawing on data from pregnant women who used substances and participated in the PRIDE-P trial, we conducted an economic evaluation of a novel behavioral intervention aimed to reduce substance use and HIV risk behaviors and improve birth outcomes. There was no statistically significant difference in main clinical outcomes between the MET-CBT and brief advice groups [13]. Our cost minimization analysis showed that the overall cost of care was similar between the two groups, and was primarily influenced by labor and delivery cost.

One strength of this study is our comprehensive measurement of costs associated with the care of pregnant women, including intervention costs, costs during the prenatal period, and maternal and newborn costs up to 3 months postpartum, as well as both hospital facility costs and physician costs. In contrast, prior economic evaluations of substance use programs for pregnant women were often limited to drug treatment costs or selected care components (e.g., neonatal intensive care unit costs) [22–25]. Few have assessed the impact of intervention on overall maternal and newborn costs. In addition, by conducting an economic evaluation in the context of a randomized trial, we were able to rigorously assess the financial consequences of the intervention as potential impact of unobserved confounding factors was minimized. In contrast, previous studies evaluating the economic impact of substance use treatment programs in pregnancy were largely based on non-randomized designs limiting their ability to draw causal inferences [22–27].

Our study suggests that health care for substance using women and their newborns is costly. This finding is consistent with other studies. For example, Whiteman et al. [28] reported that hospital facility cost alone averaged $5616 for pregnancy-related hospitalizations for women with opioid use in the U.S. during 1998–2009. Fingar et al. [29] also showed that in 2012, hospital facility cost for newborn stays related to substance use averaged $19,684, compared to $4500 for other newborns. One recent study by Goler et al [30] assessed costs of care for women receiving Early Start, an integrated prenatal intervention providing one-to-one counseling to pregnant women at risk for substance use [31]. Average cost of maternal and newborn care from prenatal until 1 year postpartum totaled $20,644 per participant, compared to $27,812 per non-participant substance user. Cost savings from medical care more than offset the program implementation cost [30]. Our cost estimates may be somewhat higher than these studies because a large proportion of participants in our study were polysubstance users and might be less healthy than patients in other studies. For example, 16.5% of our sample participants delivered preterm versus 7.7–15.4% in the various groups of substance users in Goler et al. [30]. In addition, we included costs associated with all medical services delivered during the study period, including those that were not directly pregnancy-related. This is to fully capture the impact of our study intervention which might improve participants’ health behavior and hence have broader impact on resource utilization.

Our data from the PRIDE-P trial showed that compared with brief advice, MET-CBT did not reduce overall medical costs up to 3-month postpartum. Several factors may explain this lack of a significant difference. First, pregnancy itself may substantially affect women’s health behavior, confounding and overshadowing the effect of MET-CBT. Second, many participants in our sample had relatively low substance use at baseline. Information provided in the brief advice intervention, including informing women of the adverse health impact of substance use (on both the mother and the fetus) and where additional treatment could be received, may be sufficient for these patients, making it difficult to observe the additional benefit of MET-CBT. Third, we designed MET-CBT as a one-on-one therapy to best match existing prenatal care at the study sites. This format is more costly than group therapy.

Further research assessing MET-CBT in patients with more severe substance use disorders or examining similar interventions in a group therapy setting will be informative. More research on the impact of similar psychotherapy on women’s overall quality of life will also be informative as participants may experience improved well-being and social support despite lack of significant improvement in substance use. In addition, the PRIDE-P trial suggested a trend toward a reduction in preterm birth by MET-CBT as compared to brief advice (preterm birth rate = 10% versus 20%, *p* = 0.08) although the study was not powered to assess this outcome [13]. Future studies with larger sample sizes testing the potential benefit of MET-CBT intervention in reducing preterm birth and the associated cost implications will provide additional insights.

This study has several limitations. First, our data were based on an RCT at a single study site. The findings may not be generalizable to all pregnant women who use substance or receive care elsewhere. Second, we estimated medical costs occurring within the study institution. Participants could have received care elsewhere which was not captured in our cost estimates. Third, physician cost data were unattainable at one of the study sites, precluding us from including all PRIDE-P trial participants in this analysis and reducing our statistical power for detecting differences between groups. Comparison between the two study sites also suggested that participants in our analysis were more likely to report marijuana as their primary substance (as opposed to alcohol at the other site) and a higher proportion of them were drug/alcohol abuse or dependent. However, data on hospital facility costs were available at both sites. Comparison of hospital facility costs between MET-CBT and brief advice groups at the other study site suggested no statistically significant difference either, corroborating our main findings. Finally, we were unable to perform the economic evaluation from a societal perspective as data on participants’ transportation costs and productivity loss were only collected for intervention visits but not available for other medical encounters. Likewise, we did not have data on costs associated with legal services or child custody that often occur with substance use and can be costly. Future studies incorporating non-medical costs and longer follow-up times would be informative.

## Conclusions

Substance use in pregnancy remains an important issue in obstetric care. Identifying cost-effective treatment options not only benefits the mother and her family, but also society. Our analysis of data from the PRIDE-P trial found that, compared with brief advice, a behavioral intervention integrating MET and CBT had similar effect on substance use and birth outcomes [13], and did not reduce overall medical costs up to 3-month postpartum. Continued effort to compare MET-CBT in patients with more severe substance use disorders or in different formats (e.g., group therapy) and to evaluate its impact on participants’ quality of life will further inform the potential role of behavioral interventions in managing substance use in pregnancy. Future research should also assess the potential benefit of brief advice itself in promoting abstinence in substance users.

## Abbreviations

ASI: Addiction Severity Index
DRG: Diagnosis-related group
HIV: Human immunodeficiency virus
MET-CBT: Motivational enhancement therapy with cognitive behavioral therapy
MINI: Mini-International Neuropsychiatric Interview
PRIDE-P: Therapeutic Substance Abuse Treatment in Pregnancy-1
RCT: Randomized controlled trial
STIs: Sexually transmitted infections
TWEAK: Tolerance, Worried, Eye-openers, Amnesia, K[C] Cut Down
